# Clinician and Client Reports of the Negative Effects of Neuropsychological Assessment for Dementia

**DOI:** 10.1177/08919887251407122

**Published:** 2025-12-26

**Authors:** Nikki Miller, David J. Grinter, David McGraw, Rachel Pritchett, Hamish J. McLeod

**Affiliations:** 1 Mental Health & Wellbeing, 3526 University of Glasgow, Glasgow, Scotland; 2 3077 Psychological Therapies for Older People, NHS Lanarkshire, Bothwell, Scotland; 3 1442 Older Adult Psychology Specialty, NHS Ayrshire & Arran, Ayr, Scotland; 4 Older People's Psychology Service, NHS Greater Glasgow & Clyde, Glasgow, Scotland

**Keywords:** neuropsychological assessment, client experience, negative effects, iatrogenesis, dementia

## Abstract

**Objective:**

Neuropsychological assessment (NPA) is recommended to support differential diagnosis of dementia but little is known about its impact on clients and their experience of negative effects. This study investigated clinicians’ understanding of their clients’ negative experiences and explored similarities in clinician and client reports.

**Method:**

A mixed-methods approach was employed using qualitative and quantitative data. Semi-structured interviews with clinicians, and a questionnaire for clinicians and clients were collected from NHS settings across Scotland. Reflexive Thematic Analysis was used to analyse 11 clinician interviews. Descriptive statistics were reported for the 25 clinician and 12 client questionnaires and exploratory analysis investigated associations between clinician and clients reporting of negative experiences.

**Results:**

In the qualitative analysis, three overarching themes and 13 sub-themes were identified. The over-arching themes were: assessment can produce negative impacts for clients, indirect factors can produce harmful effects, and clinicians can take action to reduce adverse effects of NPA. For the questionnaire responses, the most endorsed negative effects were the same for clients and clinicians and included feeling stressed, worried, disappointed with their performance in assessment, frustrated, critical of themselves and worried about the outcome.

**Conclusion:**

These data provide some of the first clear empirical descriptions of the negative effects of NPA as reported by both clinicians and clients. The study also identified challenges with recruiting clients who are willing to give feedback on their experience of assessment. Future studies are needed to refine the available data capture methods and to determine if the current results are replicable.

## Introduction

Reported benefits of a dementia diagnosis include increased autonomy, ability to make decisions about future care and access to services.^1,2^ Guidance from the World Health Organization^3^ emphasises improving quality of services for people with dementia and providing timely access to diagnosis. Neuropsychological assessment (NPA) helps with diagnosis and subtyping of dementia^4^ but little is known about clients’ NPA experiences and any negative effects.

Iatrogenesis, an unintended adverse outcome for a client due to a healthcare intervention, is seen in many forms of medical assessment and intervention. For example, damage to skin from the side effects of chemotherapy^5^ and false positive diagnosis in breast cancer screening leading to psychological and emotional distress.^6^ However, harm through psychological assessment and intervention receives substantially less attention.^7^ Avoidance of harm is a key ethical principle^8,9^ but there is limited understanding of how standard psychological practices may cause unintended harm nor is there guidance on how to identify and prevent this. Studies investigating clinicians’ awareness of harm have identified that 28% of American Psychological Association clinicians were unaware that clients could experience negative effects of therapy,^10^ and only 11% reported learning about negative effects during their training.^11^ Researchers have begun to acknowledge possible harms of psychotherapy,^12,13^ and measures have been developed to identify the type and occurrence of harms in practice,^14^ although this has not extended to NPA.

Studies in non-dementing working age adult populations have reported that most people find NPA useful, and that feedback on cognitive strengths and weaknesses was valued.^15-18^ Reported negative experiences suggest that around half of patients find assessments frustrating and tiring,^15^ and feedback sessions can be draining with results difficult to understand.^16^

A number of qualitative studies in varied populations of adults and older adults with neurological conditions, including possible dementia, reported benefits of NPA such as an increased awareness and understanding of difficulties.^19-24^ The relationship with the clinician was described as key to improving the experience, through supporting relaxation, facilitated coping, and increased self-esteem^19,22,24^

These qualitative studies also provided insights into negative experiences. Participants felt under-prepared for what to expect, which increased anxiety as they expected similarities to previously unpleasant medical investigations.^19^ A lack of understanding of the assessment’s purpose caused stress due to feeling pressure to perform well.^20^ Anxiety led to poor retention of information,^21^ and other reported responses included confusion, distress, frustration/irritation and anger alongside thoughts of being “stupid”, worry, fear of failure, and shame due to poor performance.^19,21-24^ Fatigue triggered by the assessment was reported by some participants.^22^ Robinson^23^ also observed that negative self-appraisals of performance affected mood and occurred in the absence of feedback.

By 2030, 78 million people are expected to have dementia. With 10 million diagnoses per year it is one of the biggest causes of disability in older people^3^ and demand on memory clinics will continue to increase. There is less emphasis on the pre-diagnostic phase of assessment in the literature but services need to understand how best to deliver person-centred care and avoid harm at this key stage of a person’s dementia journey. Only two of the studies above focussed on memory clinic populations^21,23^ and so although understanding in this specific area remains limited, qualitative exploration with clients in both studies identified negative experiences during the NPA process.

Clinicians’ awareness of potential harm and how these align with lived experience has not previously been examined. If clinicians have a limited understanding of negative effects of NPA, it could be argued that clients will not be given a comprehensive understanding of the process to allow them to give valid informed consent, a key ethical principal to which clinicians must adhere.^8,9^ This study aims to address this gap by exploring clinicians’ perspectives of negative effects from their experience of administering NPA for dementia, alongside exploring the experiences of clients undergoing NPA using a novel feedback questionnaire. The study aims to capture perspectives by utilising a tool developed by the researchers which incorporates learning from the existing literature focussed on harms in NPA, and more generally psychological therapy. At present no tool exists to capture this data in NPA and so this study offers a novel attempt to systematically capture these experiences. This dual focus on clinician insight and client experience attempts to seek greater breadth and depth in the understanding of potential iatrogenic outcomes in dementia assessment to help inform ethical and person-centred care.

## Method

### Ethics

Approval was obtained from the WoS REC (22/WS/0064) and NHS Lanarkshire Research and Development (L22004). NHS Lanarkshire’s Information Governance Department approved the use of the online questionnaire using JISC Online Surveys Design.

This study used a mixed method approach. Qualitative analysis of semi-structured interviews with clinicians, and quantitative analysis of questionnaires completed by clinicians and clients were used to understand clients’ experiences of NPA.

### Measures

Due to the lack of a suitable measure we developed a questionnaire to assess experiences of NPA for use in this study. The development of the questionnaire involved a number of steps. Firstly, an existing validated measure of negative effects in psychological therapy^25^ was reviewed to explore an acceptable format to seek these views and obtain examples of question content, which were then considered alongside expressed negative effects of NPA from the literature described in the introduction. Items included in the questionnaire were then selected and modified by combining the items deemed most relevant to reflect key elements of the NPA process from the researchers’ experience of clinical practice. Separate clinician and client versions of the questionnaire were developed, and although item content remained the same, the wording differed to reflect the viewpoint of the person answering the question (eg, the first person perspective of clients “I felt stressed” vs “Stressed” for clinicians or “Did you experience this effect at any stage before, during or after your assessment” vs “Have any of your clients experienced this before, during or after the assessment”). An additional column was included in the clinician version to acknowledge clinicians’ completion of this was reflective of a collection of client experiences (eg, “Roughly what percentage of your clients do you think this is a problem for”) and a further column included to identify if they had previously considered this negative effect as one that could be present. The next phase involved seeking both clinician and client feedback on this initial draft. The research team sought review from a Neuropsychologist colleague who worked in a different service in the health board to where the research was being conducted with the aim of ensuring face validity. Amendments were then made to item content and structure before seeking feedback from a selection of clients.

RP sought feedback on the client questionnaire from clients who were actively undergoing NPA within NHS Lanarkshire’s Psychological Therapies for Older People (PTOP) team. Four clients were asked at the end of their clinical appointment with RP to review and offer feedback. This was guided by a prompt sheet asking clients to comment on the ease of understanding of questionnaire instructions, the clarity of questionnaire items, views on the length of the questionnaire and overall impressions. Three provided feedback, and one was unable due to not having glasses available. Clinical judgment on appropriateness to seek feedback from the client and practical barriers, which included limited clinician time and cancelled appointments, limited further feedback being obtained in the timeframe. Ethical approval was then sought from the WoS REC with further amendments then made to the questionnaires to reflect requests of the panel. These included reducing the length of the questionnaire to minimise burden on the participants (reducing the item content from 49 questions to 28). Largely this involved removal of items related to their experience with their clinician (eg, “my clinician was not empathetic” or “I did not have trust in my clinician”) in favour of retaining items focussed most directly on the client’s internal experience, emotional responses and day to day impact of their NPA (eg, “I felt worried”, “I had headaches” or “I lost my driver’s license”). These final amendments were made with guidance from our Neuropsychologist colleague and final approval from the ethics panel. The final versions of the questionnaires utilised in this study are available in supplemental material file 1 and file 2. The questionnaires included 28 questions, demographic information (which was reported in a free text format), and free text option for additional comments.

### Participant Recruitment Procedures

#### Clinician Interview

A purposive sampling approach was taken with recommendations of recruitment of 6 to 10 participants by Clarke and Braun^26^ followed. Clinical psychologists delivering NPA in a Scottish Health Board (NHS Lanarkshire’s PTOP) were invited to participate via email with electronic consent forms provided. Clinicians were eligible if:

• They had completed at least one NPA with feedback within the last 12 months
• The NPA purpose was for assessment of dementia

The semi-structured interviews (Topic guide available in supplemental material file 3) were recorded on Microsoft Teams and transcribed. Following interview, clinicians were invited, via email to complete the questionnaire.

#### Clinician and Client Questionnaires

Attempts were made to recruit as many NHS Scotland Older Adult Clinical Psychologists as possible by accessing each Scottish Health Board via the Head of Older People Psychology Services (HOOPPS) group. Representatives from each Health Board were asked to share the study information with their teams by email. It is not known the exact number of clinicians that received the study information. There were no *a priori* assumptions for recruitment figures therefore an inclusive recruitment strategy was used with the aim of achieving a representative sample of views and experience.

Inclusion criteria:

• Qualified clinical psychologist
• Completed at least one NPA for possible dementia in the last 12 months

Clinicians identified eligible clients using the following criteria:

Inclusion criteria
  • Currently on a clinician’s caseload and receiving NPA for assessment of dementia (invited to participate as soon as NPA feedback had taken place) or those discharged within the last 3 months
  • English speaking
Exclusion criteria
  • Inability to provide informed consent

As the questionnaires were newly developed by the researcher, there were no *a priori* assumptions regarding recruitment numbers. Recruitment of client participants was pragmatic and determined by the number of eligible clients within the recruitment timeframe.

The capacity for client participants to consent was left to the clinical judgment of the referring clinician with clinicians asked to consider whether someone’s cognitive abilities would preclude them from completing the questionnaire. No formal review of this was undertaken by the research team and details of the participant’s clinical presentation were largely unknown to the research team. However, upon agreement with their clinician to participate in the study, NM complied with ethical expectations of informed consent ensuring that clients were able to retain information about the study procedures and cost and benefits of participation before confirming their participation. In an attempt to reduce barriers to recruitment clients were then offered a range of options for completing the questionnaire including accessing it online, completing a paper based questionnaire posted to their home with a pre-paid return envelope, or receiving assistance from a member of the research team by attending an NHS clinic site or by the researcher visiting their home. Clients choosing to attend a clinic setting were offered reimbursement of their travel expenses but no other payments were offered for participation in the study. A distress protocol was approved by the ethics board. This covered responses to distress pre and post involvement with the study, as well as support for participants during the active parts of the study.

### Data Analysis

#### Clinician Interviews

Reflexive Thematic Analysis (RTA; ref. 27) was used to explore themes in transcripts generated from clinician interviews. This approach allows patterns to be identified across transcripts, and it was chosen due to its flexibility and not being restricted to a specific theoretical base.^28^ Three transcripts were also analysed by research supervisor DG during the coding process to reduce bias and codes were discussed. NVivo9^29^ assisted with coding and theme development.

#### Clinician and Client Questionnaires

Questionnaire data were analysed using IBM SPSS (v28, 2021). Descriptive statistics were used to summarise patterns in the results and reported as frequencies. Exploratory analysis using Fisher’s Exact tests, explored associations between clinician and client endorsement of negative effects (two-tailed, *P* = .05).

### Reflexivity Statements

#### NM’s Reflexivity Statement

During the entirety of the study I was a trainee clinical psychologist working in the older adult team where the interviews were conducted during my first and third years of training. Therefore, I had my own views of possible negative experiences during NPA having worked with a number of clients throughout training and having completed NPA as part of previous roles. I entered the study with some general understanding of the process and interest in this area. The participants in the study were colleagues and so there was a pre-existing professional relationship. It was important to consider how my professional experiences and relationships could impact when conducting interviews and interpreting the data. A reflective diary was kept throughout the process to help consider any interaction of prior assumptions and views on relating to the current information being collected and the themes being developed. It was important to ensure that information was not being discounted because of any beliefs I had from my own personal experience and that views of clinicians were not being given more weight if they were in line with views I previously held.

#### DG’s Reflexivity Statement

I have worked as a clinical psychologist with older people and undertake neuropsychological assessments for dementia as a core part of my clinical practice, for 15 years. I had proposed the initial conceptualisation of the study because of my interest in critically appraising evidence-based psychological therapies and reviewing literature on negative effects in neuropsychological assessment. Whilst I analysed the interview transcripts they were anonymised and I was unaware of the participant’s identities. I had previously worked in the service where the clinicians were recruited from but had left by the time the study commenced. In analysis and interpretation, we (NM and I) used supervision sessions to discuss and debate coding, interpretations and result development, in order to help check our biases and maintain as neutral a stance as possible.

## Results

### Clinician Interviews

#### Clinician Interview Demographics

Eleven Clinical Psychologists participated in the interviews. Demographic information was aggregated to protect confidentiality. Participants were qualified between 1 and 16 years (M = 6.55, SD = 4.80) and estimations of completed NPAs was 1718 for the whole sample (range = 20 to 350, MDN = 150, IQR = 138).

### Thematic Analysis

Three overarching themes and 13 subthemes were identified and are summarised in Table 1.

**Table 1.** Overarching Themes and Sub-Themes.

Overarching theme	Sub-theme (N participants where sub-theme was expressed)
Testing can produce negative impacts on clients	Emotional experiences (11)
	Testing increasing insight into difficulties (8)
	Impact on physical wellbeing (7)
	Reduced self-confidence and self-criticism (6)
	Negative affect during feedback (10)
Indirect factors can produce harmful effects of NPA	Power dynamics and external pressures (5)
	Not understanding how clients’ history and characteristics can impact experience (7)
Clinicians can take action to reduce adverse effects of NPA	The importance of rapport and a calm atmosphere (11)
	Adapting assessments to accommodate clients’ needs (10)
	Using clinical skills to administer tests thoughtfully and retain validity (10)
	Clinical judgment on proceeding with NPA (6)
	Informed consent (9)

### Theme 1: Assessment Can Produce Negative Impacts on Clients

Clinicians did observe negative experiences through the NPA process. Emotional and physical impacts, such as body discomfort, were identified particularly related to the assessment process, and clinicians identified that there can be negative experiences during feedback.

#### Emotional Experiences

The most observed emotional impact was anxiety, with embarrassment, frustration, and stress also reported. “Test anxiety” was considered common but clinicians acknowledged that this was low-intensity and not disruptive to the validity of assessment and the wellbeing of the clients:

“I would say a mild level of anxiety would probably be present in most of the people I would see, not necessarily to clinical extent … but certainly some nervousness and apprehension about completing the testing.” (P4)

“Maybe sort of low level anxiety is a little bit more frequent, but not so to the extent that it’s disrupting.” (P1)

“You’ll get test anxiety performance, where people, it’s almost like a school test they get worked up. You can see it in their face, you can see in the body language and it’s usually up the clinician at that point to pick up on that and say do you want to pause?” (P6)

Clinicians noted that negative affect was an understandable part of the process and there is an unavoidable element to this given the context of the situation:

“Yes, it will be upsetting but ultimately, people go through that, though, to get to an answer to help any kind of interventions that might be there. So while there’s discomfort, perhaps some distress, it’s a necessary evil in the overall process. As long as the clinicians attuned to their patient, you should be able to manage that in a session by session basis.” (P6)

“I suppose you know when you’re doing an assessment process that has the potential at the end of it to tell someone that they have a terminal diagnosis I think we have to as clinicians recognise that is that is very likely to cause someone distress and we cannot mitigate that, and nor should we.” (P11)

#### Assessment Increasing Insight Into Difficulties

Clinicians described how the process of assessment can bring difficulties to the forefront that have been able to be avoided in daily life forcing clients to confront a deficit.

For some clients it was acknowledged that assessment contributed to them developing insight about a problem:

“That kind of frustration and feeling they should be doing better than they are, and it kind of I guess it brings people’s difficulties to the fore and even if they haven’t, maybe had much insight into them in their day-to-day lives, when you put them in the testing situation that maybe highlights them more so.” (P4)

“It’s quite confronting in that it’s right there in front of you, like I can’t remember this list, I can’t remember that picture. I don’t even remember you asking me about the picture. You know, there’s that kind of very, I think we can sort of avoid that in day-to-day life by avoiding things that are hard or whatever or just kind of playing to our strengths.” (P9)

For other clients assessment was preventing them from continuing to use self-protective methods to avoid acknowledging their difficulty:

“The carer was like, ‘you’ve definitely got a problem with your memory’ and they were like, ‘no, I don’t’. But it came out through the testing process that actually they knew they did. They just didn’t want to admit it. So that kind of not being able to live in denial really being confronted with it. And they were quite happy in denial. Like, that was good for their self-esteem.” (P2)

#### Impact on Physical Wellbeing

There was an acknowledgment that the assessment environment can fail to provide the optimum conditions for clients:

“It is also important in terms of a person’s comfort and now comorbidities with older people, chronic pain, arthritis, osteoporosis, emm, poor vision poor hearing, toileting issues … because of lack of rooms, that are adequately resourced, we will find ourselves cramped in small rooms using tables that are at knee height where older people need to bend over.” (P6)

“I think there was a physical impact, Just the need to sit for a long period of time and attend, um, physically had an impact on pain levels.” (P2)

And an awareness that assessment can have an impact on clients’ physical health beyond the appointment and interact with comorbid conditions:

“A huge comorbidity seems to be chronic pain. So when people go home, they’ll be absolutely shattered and probably usually do nothing for the rest of the day.” (P8)

“Sleepless nights before the test because he was just sort of, you know, because he knew he was going to find it difficult.” (P1)

#### Reduced Self-Confidence and Self-Criticism

Developing insight and increased awareness of difficulties was observed to impact on clients’ confidence and self-critical comments were common:

“And you can see the frustration and you can see also the element of self-criticism that comes into that. Sometimes there’s a commentary during testing where someone will say something like “that was stupid”, you know, or “that wasn’t very good”. (P11).

And a knock-on effect of this was considered in terms of clients’ mood and their view of themselves:

“It’s particularly difficult for people who have good insight into their difficulties but do have significant difficulties and have previously been very independent, high functioning people, it can just really knock their kind of sense of self, sense of self-worth.” (P3)

“‘I think we can sort of avoid that in day-to-day life by avoiding things that are hard or whatever or just kind of playing to our strengths, but when you’re confronted with it and paper in a test, it can be quite upsetting” (P9)

#### Negative Affect During Feedback

Clinicians were aware that feedback appointments can elicit negative affect in anticipation of dementia diagnosis and acknowledged this is an expected part of the process however some factors may increase negative affect.

Some clinicians acknowledged that clients who lack insight into their difficulties can find feedback sessions difficult as a diagnosis comes as a surprise:

“It’s more common with Alzheimer’s clients to have poor insight into their memory deficits. It’s sometimes they could seem a touch taken aback when you’re explaining the severity of their memory impairment.” (P1)

“There was this gentleman who himself lacked insight into his difficulties so the diagnosis, just didn’t marry up with his reality, and so he had quite an extreme reaction. He stated that he was going to end his life … and once he’d calmed down from receiving the diagnosis, there was no intent there.” (P2)

Another situation where clinicians observe distress in clients during feedback is when they are not given a diagnosis and this is what they were expecting because they feel it helps explains their difficulties, and so they are left unsure about the cause of their difficulties:

“The ones that come to mind, who have maybe not been happy with the outcome, not agreed with the outcome being a bit frustrated, have been the ones who’ve not got a diagnosis. And it’s a wait and see. So it’s mostly it’s when they’re lived experience doesn’t quite map on to how I’ve put it under the diagnostic criteria.” (P7)

“The testing showed that they were actually performing really well and that obviously caused a lot of anxiety. So the testing, they didn’t get the diagnosis that they had related to and wanted.” (P2)

### Theme 2: Indirect Factors Can Produce Harmful Effects of NPA

There are two sub-themes of factors that could indirectly negatively impact the client experience.

#### Power Dynamics and External Pressures

The power imbalance in clients’ relationships and the impact it could have on informed consent and their engagement in NPA was highlighted:

“Then you have ones who will say yes to anything a doctor says so you worry about power differentials within that and you think, are they fully consenting and engaged in a process?” (P6)

“You feel the expectations of your family and you feel the expectations of your therapist sitting in front of you, who is an authority figure. And often it’s the authority figures I had you know that trauma happened through to some extent. Not in every case, but in some cases, it can be quite a triggering process so that’s something to sort of to think about and kind of be mindful of.” (P10)

Expectations of family or doctors involved in the client’s care may influence their decision to attend:

“Often the clients themselves don’t have much of an expectation, they come along because they’ve been sent by their psychiatrists and their families think they should come along.” (P10)

#### Not Understanding How Patients’ History and Characteristics Can Impact Experience

The impact of past educational experiences on clients’ current feeling about engaging with NPA was acknowledged:

“Nobody wants to do badly on it. It feels like a test. And there’s like, I think there’s also, like, people who maybe are a bit insecure about their intelligence or their, like, performance at school if they didn’t, if they weren’t particularly academic at school or they struggled with tests. I think that can also be quite a source of anxiety and stress about not doing well.” (P9)

Clients’ perceptions of themselves or concerns about how other people will view them was considered to contribute to the experience being more anxiety provoking:

“Long standing beliefs about self-esteem or you know, educational attainment, or being judged by other people. So yeah, a lot of people can find it very anxiety provoking.” (P3)

### Theme 3: Clinicians Can Take Action to Reduce Adverse Effects of NPA

Clinicians often actively make decisions to ensure clients had a good experience and they mitigate potential risk of harm during client interactions.

#### The Importance of Rapport and a Calm Atmosphere

Clinicians talked about the importance they put on providing a relaxed, supportive, and friendly atmosphere.

Some clinicians mentioned the importance of investing in building a collaborative relationship with the client rather than *“… just kind of getting people into the appointment and jumping straight into testing, maybe, kind o,f checking in with them and having a bit of chat.” (P2)*. The importance of this for ensuring optimal assessment performance and thus assessment validity was considered:

“You just want them to be as calm as possible and have less cortisol running around their body. Because then that means that their frontal lobes won’t shut off, which is something that you’re trying to study.” (P8)

Clinicians believed that positive rapport can help the clients have a better feedback experience:

“I think with patients, with whom you’ve had perhaps a better alliance, you do find that at the end at the feedback appointment they sort of perhaps they feel a bit safer in receiving whatever news they get.” (P10)

“You can try and have a wee bit of banter with people or a bit of humor with people and I think it really helps to build up that that relationship because hopefully then by the time it comes to the feedback, they feel that they can, they can trust you in, in what you’re saying to them,” (P4)

#### Adapting Assessments to Accommodate Clients’ Needs

Clinicians were considerate of clients’ physical and sensory needs when deciding where to offer appointments and how to adapt the session structure to limit any negative impacts and get the best performance was acknowledged:

“I think we’re quite flexible in how we offer things. So if people were struggling to travel, as well in my area because it’s quite rural, that if they couldn’t travel, we would go to them.” (P2)

When meeting the client, clinicians explored physical health difficulties that could be exacerbated by NPA and considered how they can set up appointments to maintain comfort and retain the validity.

“If someone has um, particular physical issue, chronic pain, or is going to be uncomfortable for them to, I would usually be assessing that before I would start. “How long do you feel comfortable sitting?”, you know, and “when do you take your pain medication?”. Things like that to try and arrange it so that you’re getting the best conditions.” (P11)

Clinicians described taking a person-centred approach to understand the clients’ needs and adapt sessions to ensure there is no impact on their experience or validity of testing:

“I had one lady I worked with who had quite significant respiratory issues. Emm and when we were doing the testing the testing kind of increased anxiety which led to an exacerbation in her respiratory issues and so we, we had to kind of set up a bit of a plan of how to pace the session, shorter sessions, we can spread out over more appointments and have more regular breaks during testing.” (P4)

#### Using Clinical Skills to Administer Assessments Thoughtfully and Retain Validity

Clinicians described the importance of considering which assessments to administer and when, and how to administer these in a way that engages the client whilst minimising the possibility of assessment eliciting a negative emotional experience.

Clinicians explained they provide encouragement rather than giving no feedback, to celebrate clients’ effort but avoiding telling them how they are performing to retain assessment validity:

“Sometimes I’ll give slightly arbitrary just little bits of something just like, ‘Yep, that’s great. Yup. Well done.” And it doesn’t necessarily literally mean like, “hey, you just performed very well above the norms”. It just means well done for like, trying your best at that.” (P1)

Considering the clients presentation at the time of assessment helps clinicians to consider what assessments to administer and in what order. Clinicians described being attuned to how their clients are responding to the assessment process and offering reassurance if necessary:

“I do think that like test selection and the order can have an impact at times. So if people are particularly anxious not doing like a really difficult test straight off the bat, so maybe kind of easing them in.” (P2)

“I probably just support it quite a lot of like validating you know spotting the frustration and labelling it and validating it early on. I always say to people if you get 100% and come out of here feeling that you’ve done really well, I’ve picked the wrong tests.” (P7)

#### Clinical Judgment on Proceeding With NPA

From the point of referral to assessment clinicians considered the value of undertaking NPA with the client and remained attuned to changes which might suggest continuing with NPA was not useful. Clinicians attempt to proactively reduce anxiety levels when they are identified:

“I have had a few cases where I’ve done like kind of relaxation breathing techniques with before we even started testing and kind of that was the only way that I could get an accurate valid test performance.” (P2)

Clinicians are conscious of the confounding impact that anxiety can have on cognition and assessment performance:

“So people do get anxious about their performance at times. So, it’s not nice to see someone feeling anxious while doing tests. I discontinue at that point. Because one, it’s unethical and two you’re not assessing organic abilities, you’re assessing how performance anxiety effects cognitive performance.” (P5)

“If you are thinking more neuropsychologically then if someone’s anxious then they can’t take in information and they’re working memory will be impacted by that. And if someone’s working memory’s impacted, then everything else is because attention is kind of the centre of a lot of different things. And it means that cause instructions are quite complicated, so if they can’t be retained, you’re not necessarily measuring the thing that you are setting out to measure and you can’t really rely on the neuropsych[ological assessment].” (P8)

#### Informed Consent

Clinicians invest time in ensuring clients are fully informed and consent to the process and make attempts to ensure this information is remembered:

“I’ll have that conversation first appointment, I’ll give them a written summary away and then we’ll kinda revisit that question at the start of pretty much every appointment.” (P3)

“I do prediagnostic counselling, many times. I circle around because I think perspective, insight, retention. I don’t think it’s, I don’t think it’s something you can do in one conversation. I don’t think it’s accurate. And I think, the educational component needs a bit of time to digest and to think through. And the layers of protective defences and, and so on, I think you need to give, give folks a chance.” (P7)

Clients’ choice remains at the forefront of clinicians’ decision-making and consent was revisited throughout assessment:

“They shouldn’t be stepping in to the unknown with any uncertainty. They should know what they’re going into with their eyes open and aware, even if they forget. The next day, the next time I would see the patient I’d review really briefly what we discussed, what we agreed, were they still OK with that. So giving choice. Choice, choice, choice, ensuring that they want to do it is just so important.” (P5)

“I mean, people, people have rights to decline treatment and even if it is in their best interest and they still have a right to decline. And I think we don’t emphasise that enough with patients.” (P10)

Clinicians viewed it as their responsibility to ensure clients were fully informed about the process and expressed a responsiveness to retaining clients’ choice throughout the process.

### Clinician and Client Questionnaires

#### Sample Characteristics

Twenty-five clinicians completed the questionnaire over a 5-month recruitment period. Participating clinicians had been qualified between 1 and 21 years (M = 8.5, SD = 5.5).

**Figure 1.**
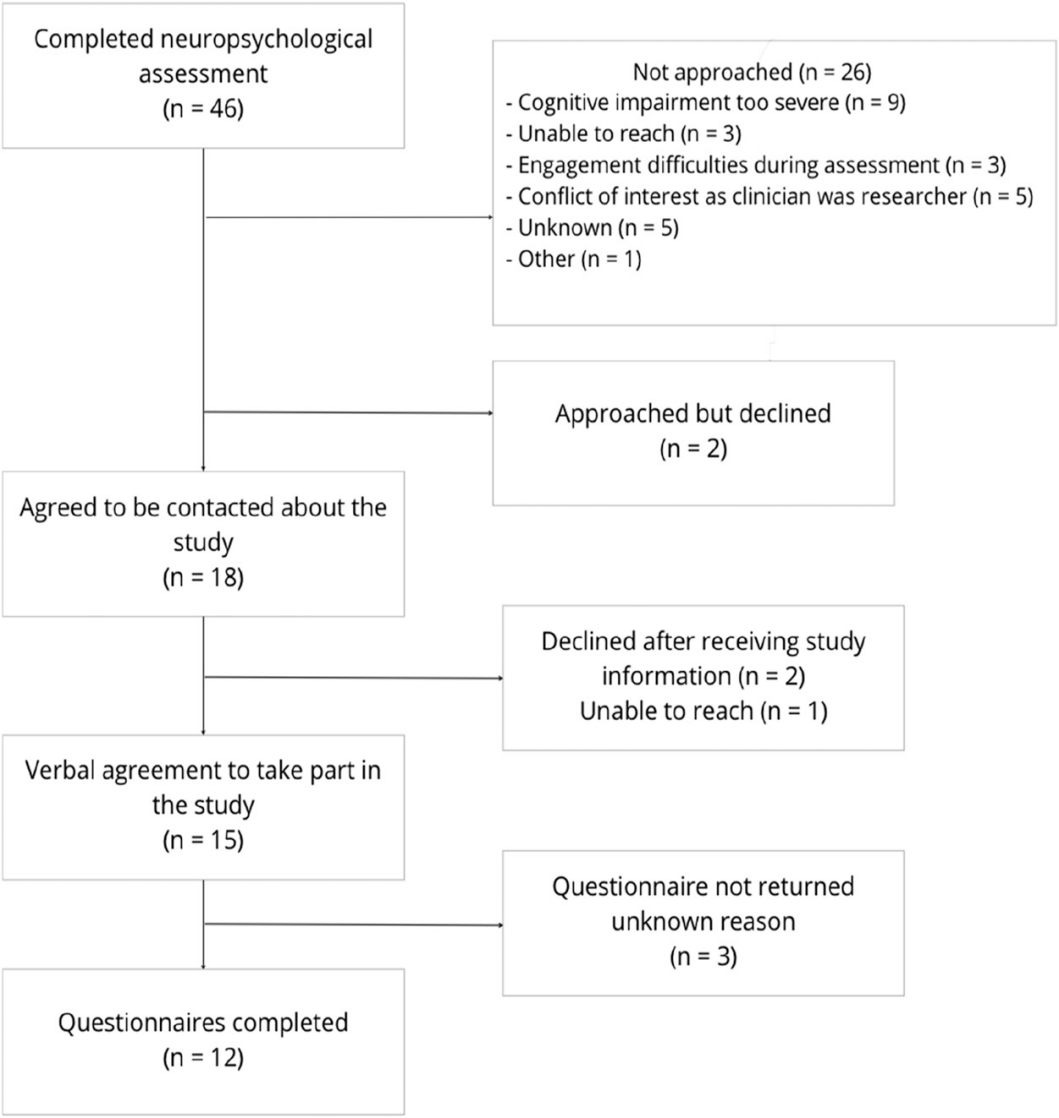
Client recruitment flowchart.

During the recruitment process, described in Figure 1, 46 clients were estimated to have received NPA feedback. Twelve questionnaires were returned, representing 26% of the clients receiving NPA. Table 2 summarises the demographic information which was requested in a free text format with no pre-existing options provided.

**Table 2.** Client Demographic Information.

Demographics	Frequency (%)
Age (N = 12)	
Range: 62-81	
M: 71.5	
SD: 5.89	
Sex	
Female	7 (58.33)
Male	5 (41.67)
Diagnosis after assessment	
AD	2 (16.67)
VD	2 (16.67)
MCI	4 (33.33)
Medication/psychiatric related CI	1 (8.33)
No diagnosis	3 (25.00)
Ethnicity	
Scottish	3 (25.00)
British	1 (8.33)
British White	2 (16.67)
White	2 (16.67)
Unknown	4 (33.33)
Mental health difficulties	
Anxiety	1 (8.33)
Depression	1 (8.33)
Mixed anxiety/depression	1 (8.33)
None	5 (41.67)
Not reported	4 (33.33)
Physical health difficulties	
Diabetes	2 (16.67)
Osteoarthritis	2 (16.67)
Arthritis	1 (8.33)
Hypertension	2 (16.67)
Epilepsy	1 (8.33)
Diverticulitis	1 (8.33)
None	5 (41.67)
No health information reported	3 (25.00)

Abbreviations: AD, Alzheimer’s Disease; VD, vascular dementia; MCI, mild cognitive impairment; CI, cognitive impairment.

#### Missing Data

Missing data and response percentages for each question are reported for transparency in supplemental material file 4. Four of the returned client questionnaires had substantial missing data and for the whole dataset, missing responses ranged from 16.7% to 41.7% for each question. All returned data is included in the results to ensure the breadth of client experiences were included. The impact of this on representativeness of the sample, feasibility of data collection and possible explanations for high rates of missing data will be considered in the discussion.

#### Negative Effects Reported by Clinicians and Clients

The top six negative effects were the same for clinicians and clients (Table 3). For clinicians, 100% endorsed disappointment with performance on the tasks, frustrated with self, critical of self and worried about the outcome of assessment. Stress and worry were observed by 96% of clinicians. For clients, worry about the outcome of assessment was reported by 83.3%, worried and disappointed in performance on tasks reported by 66.7, stressed and frustrated was reported by 58.3%, and critical of self by 41.7% clients.

**Table 3.** Clinician and Client Responses for Presence of Negative Effects With Fisher’s Exact Test Results, Odds Ratios and Confidence Intervals.

	Endorsement of negative effects		Fisher’s exact test						
	Clinician N (%)	Clients N (%)	*P* value	Odds ratio	95% CI lower-upper	Clinician N		Client N	
						Yes	No	Yes	No
Q1 Stressed	24 (96%)	7 (58.3%)	.061	0.097	0.009-1.088	24	1	7	3
Q2 Worried	24 (96%)	8 (66.7%)	.190	0.167	0.013-2.093	24	1	8	2
Q3 Hopeless	14 (56%)	1 (8.3%)	.047*	0.098	0.011-0.908	14	11	1	8
Q4 Sad	17 (68%)	3 (25%)	.116	0.235	0.047-1.190	17	8	3	6
Q5 Disappointed in performance on the tasks	25 (100%)	8 (66.7%)	.265			25	0	8	1
Q6 Frustrated	25 (100%)	7 (58.3%)	.242			25	0	7	1
Q7 Critical of self	25 (100%)	5 (41.7%)	.010*			25	0	5	1
Q8 Suicidal ideation	13 (52%)	0 (0%)	.012*			13	11	0	8
Q9 Irritable	19 (76%)	2 (16.7%)	.015*	0.105	0.017-0.666	19	6	2	5
Q10 Angry	15 (60%)	1 (8.3%)	.039*	0.095	0.010-0.897	15	10	1	7
Q11 Embarrassed	21 (84%)	4 (33.3%)	.034*	0.152	0.028-0.830	21	4	4	5
Q12 Disempowered	12 (48%)	2 (16.7%)	.416	0.361	0.061-2.146	12	13	2	6
Q13 Stupid	21 (84%)	4 (33.3%)	.074	0.190	0.033-1.097	21	4	4	4
Q14 Confused	23 (92%)	4 (33.3%)	.007*	0.070	0.010-0.491	23	2	4	5
Q15 Worried about the outcome	25 (100%)	10 (83.3%)	.099			25	0	10	2
Q16 Physically tired	23 (92%)	1 (8.3%)	<.001*	0.012	0.001-0.158	23	2	1	7
Q17 Mentally drained	23 (92%)	3 (25%)	.004*	0.052	0.007-0.399	23	2	3	5
Q18 Headaches	12 (48%)	3 (25%)	.458	0.464	0.097-2.217	12	13	3	7
Q19 Problems with sleep	19 (76%)	3 (25%)	.082	0.189	0.035-1.038	19	6	3	5
Q20 Strain on family relationships	20 (80%)	1 (8.3%)	.001*	0.036	0.004-0.361	20	5	1	7
Q21 Lost out financially to attend appointments	9 (36%)	1 (8.3%)	.225	0.222	0.024-2.074	9	16	1	8
Q22 Gave up significant amounts of time to attend	17 (68%)	2 (16.7%)	.025*	0.134	0.023-0.799	17	8	2	7
Q23 Lost driver’s license	19 (76%)	0 (0%)	<.001*			19	6	0	8
Q24 Did not understand the purpose of assessment	20 (80%)	1 (8.3%)	.001*	0.036	0.004-0.361	20	5	1	7
Q25 Did not understand the results of assessment	12 (48%)	1 (8.3%)	.108	0.155	0.017-1.450	12	13	1	7
Q26 Was not made aware of the risks involved in assessment	1 (4%)	2 (16.7%)	.113	9.600	0.723-127.532	1	24	2	5
Q27 Did not feel prepared for what the assessment involved	3 (12%)	2 (16.7%)	.591	2.095	0.289-15.191	3	22	2	7
Q28 Waited too long to receive feedback	7 (28%)	1 (8.3%)	.403	0.321	0.034-3.064	7	18	1	8

*Significant result (at *P* < .05) indicating a difference in level of endorsement by clinicians and clients.

#### Summary of the Most Endorsed Negative Effects

**Figure 2.**
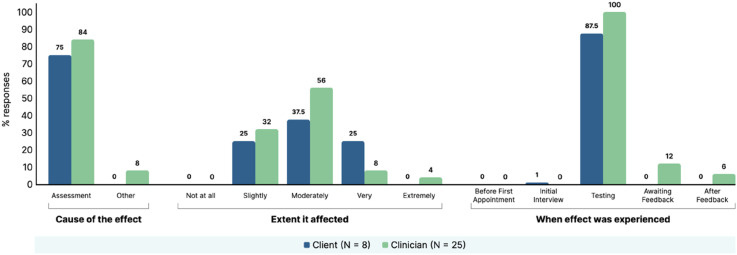
Summary of clinician and client percentage responses for question item ‘Disappointment with performance on the tasks’.

**Figure 3.**
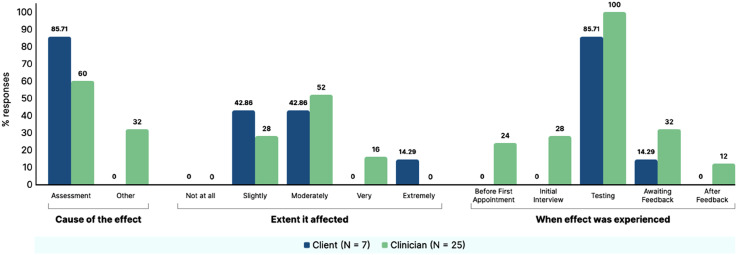
Summary of clinician and client percentage responses for question item ‘Frustrated with self’.

**Figure 4.**
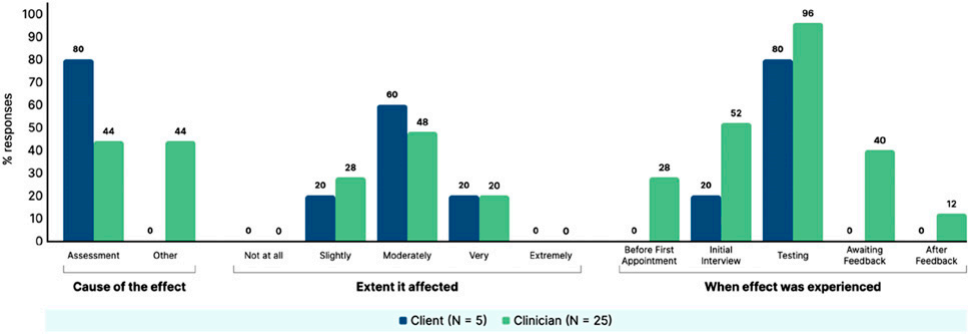
Summary of clinician and client percentage responses for question item ‘Critical of self’.

**Figure 5.**
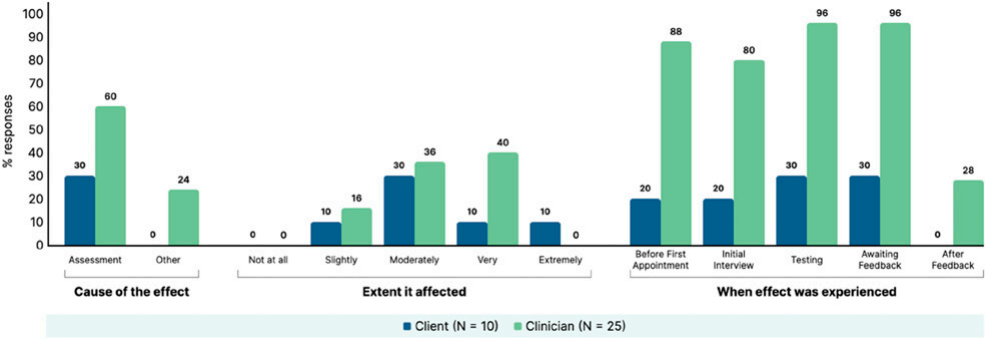
Summary of clinician and client percentage responses for question item ‘Worried about the outcome of assessment’.

**Figure 6.**
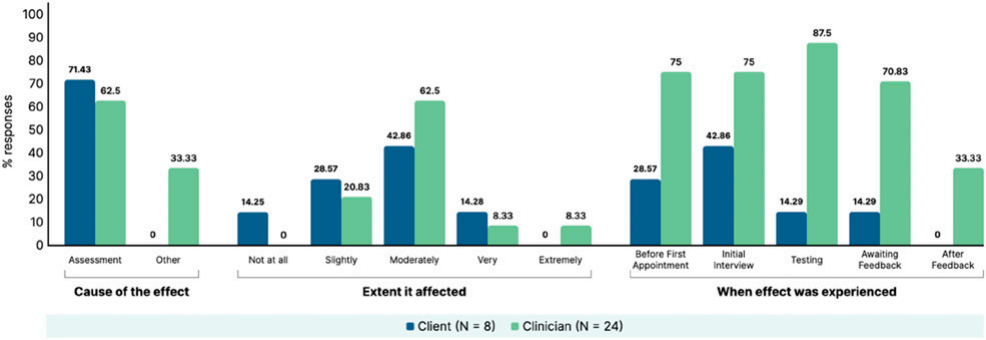
Summary of clinician and client percentage responses for question item ‘Stressed’.

**Figure 7.**
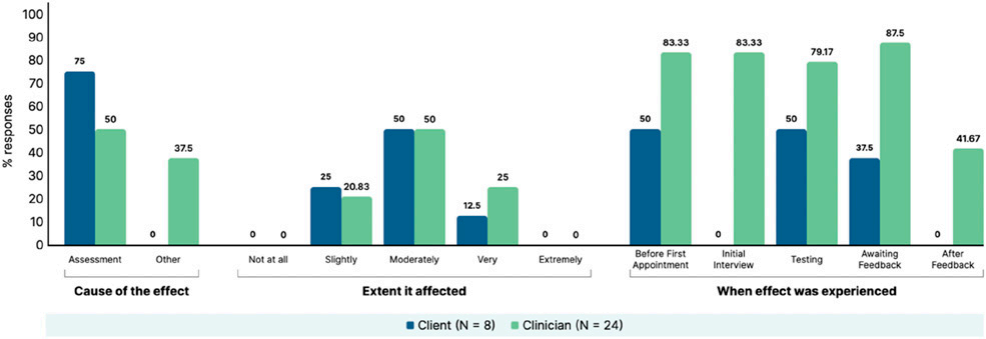
Summary of clinician and client percentage responses for question item ‘Worried’.

For those who endorsed the presence of an effect, the questionnaire also asked participants if they thought the effect was due to the assessment itself or other circumstances, the extent the effect impacted on the client and at what point during the assessment process the effects were observed (data summarised in supplemental material file 4). A visual summary of the results from these six most endorsed effects are presented in Figure 2–7.

#### Comparison of Clinician and Clients Reporting of Negative Effects

Fisher’s exact tests compared reporting of negative effects between clinicians and clients (Table 3). Fifteen of the question items were not significant which suggests that clinicians and clients endorsed the presence of these effects to the same degree. Thirteen question items were significant indicating a difference in the levels of endorsement between clinician and clients. For all significant results, these items were endorsed by a greater percentage of clinicians than clients.

## Discussion

### Clinicians Understanding of Negative Effects

Three key themes were identified: (1) assessment can produce negative impacts for clients; (2) indirect factors can produce harmful effects of NPA, and (3) clinicians can take action to reduce adverse effects of NPA. All negative effects stated in the questionnaire were endorsed by at least one clinician, indicating all the effects have been observed to a greater or lesser extent. The most endorsed effects were stress, worry, disappointment with performance, frustration with self, critical of self and worry about the outcome. These results are consistent with clients’ experience of NPA in a range of populations with neurological conditions including dementia.^19,21-24^ The results indicate that within NPA for assessing dementia, clinicians are aware of at least some potential negative effects. This is a contrast to psychological therapy research where only 28% of clinicians reported awareness of harms.^10^ With only 11% of clinicians reporting that they learned about negative effects during training,^11^ there is a need for explicit teaching on possible harms from psychological practice and how clinicians navigate these.

This study supports previous claims,^21,23^ that assessment increases awareness of difficulties which in turn impacts self-confidence and increases self-criticism. As contact with the assessing clinician often ceases after NPA concludes, it is unknown whether these impacts persist, and whether clinician intervention can minimise the possibility of lasting negative effects.

Robinson^23^ reported that clients’ self-perceptions of their performance influenced their affect, and self-judged poor performance triggered negative emotional experiences and worry about declining cognition. These self-appraisals are more common in the absence of performance feedback which is standard procedure in NPA administration. In this study clinicians spoke of praising efforts throughout assessment when they cannot give feedback and validating frustration when it arises. Clinicians’ interpersonal skills and ability to remain attuned to clients appear critical for minimising possible harms and is reflected in NPA guidance^30^ which states the clinicians’ should be aware of the factors that are detrimental to clients’ performance.

The importance of a good rapport, as identified in previous studies,^19,22,24^ was emphasised by clinicians who acknowledged their role in creating an environment that was conducive to a valid assessment. Clinicians acknowledged the presence of power dynamics and the influence of external factors on clients’ attendance at appointments. It was clear from clinician reports that gaining informed consent and ensuring clients felt involved in decision making was a key part of the assessment. Data on older peoples’ preferences for involvement in decision making in health care is contradictory with older adults wanting to be active participants in decisions^31^ or more passive and placing trust in their doctor’s opinion.^32^ Clinicians conducting dementia assessment are in a position of power and encouraging clients to be active participants in decisions about engaging in the process is crucial.

### Negative Effects Reported by Clients

The most endorsed effect for clients was anxiety about the outcome of the assessment, followed by general worry, disappointment with task performance, stress, frustration and self-criticism.

Recruitment difficulties and high rates of missing data necessitate cautious interpretation of these results. Four of the questionnaires had significant missing data. Missing data rates were higher for all in the follow up questions which included the extent the effect impacted them, when the effect was apparent during the NPA process and whether it was due to the assessment specifically. These questions may not have been as well understood as the initial yes/no presence of negative effects. Level of cognitive impairment has been found to impact data quality^33^ and response patterns in questionnaires can be associated with risk of future dementia diagnosis.^34^ Levels of cognitive impairment in this study may have influenced completion of the questionnaire and future researchers should ensure accessible methods of collecting feedback. Factors that could improve data quality such as support from a caregiver were not investigated in this study, but it is feasible that reducing the cognitive burden of the questionnaire might improve engagement. NM supported two participants upon their request to complete the questionnaire and neither case had any missing data. For those who returned the questionnaire by post it is unknown what level of support they had from others to complete the questionnaire but it could be anticipated that embedding carer/family or staff support in obtaining feedback would likely help reduce missing data.

Representative recruitment of people with dementia in research has faced challenges, with prevalence rates, specific subtypes and comorbidities not accurately reflected in participant samples.^35^ Key challenges have included ill health, adjustment to diagnosis and study burden,^36^ and extended timeframes to successfully recruit were proposed as needed.

### What Similarities and Differences are Noted in Client and Clinician Reports of Client Negative Effects?

As this is early phase research, the descriptive statistics and clinician-client rating comparisons should be interpreted with caution. However, it was of interest that the top six most endorsed effects were the same between clinicians and clients. There were similar agreement levels for some negative effects but significant differences in others and larger recruitment numbers would be required to make inferences about these trends.

Clinicians tended to report a higher overall frequency of negative effects than clients. Several explanations may account for this divergence. Clinicians have a broader frame of reference for negative experiences due to their cumulative experience across multiple clients often over many years. Whereas clients were drawing only on their own experience. It is also possible that the clients who agreed to participate were less distressed, had a better relationship with their clinician and were more motivated to engage, representing a subgroup that was likely less negatively impacted and therefore resulting in an underreporting of negative effects. The potentially transitory nature of some negative effects, and an allowance of up to 3 months post assessment to participate, may have meant that negative effects that had been present at the time of assessment were not remembered at the time of reporting by those with memory difficulties.

### Strengths and Limitations

This study is the first to investigate clinicians’ views of negative effects of NPA for dementia. The exploration of views of clinicians and reports from clients has added to the limited understanding of negative effects from NPA. Another strength was the heterogeneity of diagnoses, despite low recruitment numbers, reflective of the diversity seen in clinical practice.

There are several limitations impacting the generalisability of the results. Recruitment of client participants was low and missing data was relatively high so cautious interpretation of the results is required.

Although feedback from clients was sought at the study development stage, formal acceptability and feasibility investigations have not been undertaken. This would be beneficial given the difficulties with recruitment and data quality. The basis of the questionnaire was the Negative Effects Questionnaire (NEQ; 25) which has excellent internal consistency (α = 0.96) and has a reliable factor structure, but reliability and validity of the present neuropsychology variant needs undertaking as the NEQ is not reflective of experiences expected during NPA and has not been validated in populations where cognitive difficulties are prevalent.

In this study and abiding with the stipulations agreed with the Ethics Committee, limited clinical information regarding participants was available to the researcher that would allow a wholly accurate interpretation of the data obtained but a number of possibilities have been considered as factors that could have impacted engagement and completion of the questionnaires.

Given the variability of the population being studied and vast range of abilities expected in those undergoing assessment due to cognitive changes it would be anticipated that some participants may have difficulties in their ability to interpret and respond to a questionnaire of this kind due to the demands on language, memory and visual processing skills. Clinical observation obtained from RP during client feedback on initial drafts of the questionnaire offer some insight into possible difficulties in client completion of the questionnaire, with difficulties in understanding the questionnaire and task instructions reported in the client who from clinical judgment was more cognitively impaired. It is also of note that the delay between assessment and data collection varied and this timeframe was not recorded by the researchers but it is acknowledged that this could have had an effect on rates of item completion and accuracy of recall of negative experiences. This is an unfortunate but unassailable issue when exploring experiences of people with memory problems, however, it may have introduced inaccuracies into the data via forgetting, misremembering and confabulation. Guidance was offered to referring clinicians to retain representativeness of the sample and ultimately a judgment was made by the referring clinician regarding the suitability and appropriateness of clients to be able to complete the questionnaire and reflect on their experiences.

Clinicians are often the gatekeepers to clients accessing research. There are known bottlenecks in recruitment within clinical research with clinicians recruiting their clients, with clinician work pressures, clinician attitudes towards research, conflicts of interest and concerns about participant burden being limiting factors.^37-39^ Clinician gatekeeping has been argued to have adverse ethical implications such as denying clients’ self-determination, preventing representativeness of samples, and creating biases in understanding effectiveness of interventions.^40^ In this study clinicians’ referral decision making is not well understood but client distress and level of cognitive impairment were expressed as reasons for not referring. It could therefore be speculated that clients who experienced greater distress may have potentially reported a greater number of negative effects, but their experiences were not captured during this study as distress presented as a barrier for being approached by clinicians at the recruitment stage. Additionally, those who were referred who had negative affect may have been less willing to engage with research, or those given a diagnosis may not have felt the time was right to engage in research (as noted by Field et al, 2019). Whilst the research team attempted to engage the clinicians with the study it is recognised that to improve the chances of obtaining greater recruitment and representative samples having closer working relationships and embedding a culture of research within healthcare settings may have been needed.

The ethnic profile of the sample was consistent with census data indicating Scotland to be predominantly White (96.02%; ref. 41) but is not as diverse as would be seen in other settings. Thus, the views identified in this study cannot be said to be representative of those from minority ethnic groups, as cultural factors can impact client experience, and clinician interpretation, of NPA.^42^ Culturally shaped beliefs about ageing, cognitive change and help seeking will impact how individuals perceive the purpose and usefulness of NPA.^43^ Due to cultural norms around emotional expression^44^ the attempt of the questionnaire to seek possible negative experiences may not capture experiences of certain cultural groups due to differing interpretations and expressions of distress. The ethnicity and cultural background of the researchers were largely aligned with those of the participants, which may have influenced the assumptions embedded in the questionnaires used, particularly within the phrasing and selection of items. This shared cultural context may have been beneficial for the population studied but also introduced bias, highlighting the importance of further research to explore the relevance and applicability of feedback tools across diverse cultural settings.

### Clinical Implications & Future Directions

Notwithstanding the limitations described above, the interviews and questionnaires identified commonly experienced negative effects. It is increasingly recognised that the views of people with lived experience should be included in studies, and pre-diagnostic counselling (PDC) is no different^45^ It should be good clinical practice to explain to clients during PDC the possible negative experiences they might encounter to ensure they can give informed consent. The views of clinicians and clients in this study contribute to an enhanced understanding that could be incorporated into an update of PDC guidance^46^ to ensure inclusion of recognised negative effects to better inform future clients and guide clinicians’ discussions during this process. In the clinic, the assessment administrators could adapt practice to include check-ins regarding iatrogenesis into their protocols on a session by session basis or could provide written information on this to help remind those with memory impairments, for example.

Improving recruitment success and response rates should be a key aim of future research. This study highlighted some of the challenges in obtaining client experience data and these difficulties in clinical practice prevent older adult services from effectively evaluating their service delivery. It is important for clinicians to ensure accessibility of feedback methods rather than exclude clients from this opportunity entirely. The questionnaires developed for this study were an attempt to capture the broad range of possible experiences in an area that currently has little attention in the literature, and as yet where no validated measure to seek this information exists. Given the noted difficulties with recruitment and missing data using this method, further refinement and piloting of this measure are warranted. Larger sample sizes in more diverse samples would be important to strengthen this questionnaire’s clinical utility and guide revisions to enhance accessibility and reliability. Including feedback from more diagnostically and culturally diverse samples would allow exploration of factors that may shape perceptions of NPA. The small sample size and missing data in this study limited the ability to explore possible subgroup differences, such as those related to the diagnostic category of clients or clinician characteristics, which may influence how negative effects are perceived and reported.

It is also feasible that clients did not endorse particular question items because the words used were not those they would choose to describe their difficulties. Future research on negative effects of NPA should check the idioms clients and families use to describe their experiences cross-culturally and should also determine preferred ways of sharing their feedback. Parry and colleagues^47^ acknowledged the problem in psychotherapy harm literature is that improved understanding has been hindered by a lack of consistent language and definition of what constitutes harm, and additionally a lack of reporting harms within published research.

Collecting information from clients throughout the process of assessment, rather than retrospectively would improve accuracy of the data and might also reduce the gatekeeping of referrers, as they might be more comfortable recruiting people before they have given diagnoses, given the emotional impact this has on clients. Consideration should also be given to how family and carers could be included, where appropriate, to help elicit and communicate feedback on experiences as this would likely enhance data quality.

### Conclusions

This study offers new insights into the negative experiences of clients undergoing NPA for differential diagnosis of dementia as observed by clinicians and reported by clients. Future research would benefit from examining acceptability and feasibility of this questionnaire by recruiting a larger sample of participants, better operationalising the definition of negative effects to ensure cross-cultural applicability, and improving accessibility of feedback measures in older adult populations with cognitive impairment.

## Supplemental Material

Supplemental Material - Clinician and Client Reports of the Negative Effects of Neuropsychological Assessment for Dementia Supplemental Material for Clinician and Client Reports of the Negative Effects of Neuropsychological Assessment for Dementia by Nikki Miller, David J. Grinter, David McGraw, Rachel Pritchett, and Hamish J. McLeod in Journal of Geriatric Psychiatry and Neurology

Supplemental Material - Clinician and Client Reports of the Negative Effects of Neuropsychological Assessment for Dementia Supplemental Material for Clinician and Client Reports of the Negative Effects of Neuropsychological Assessment for Dementia by Nikki Miller, David J. Grinter, David McGraw, Rachel Pritchett, and Hamish J. McLeod in Journal of Geriatric Psychiatry and Neurology

Supplemental Material - Clinician and Client Reports of the Negative Effects of Neuropsychological Assessment for Dementia Supplemental Material for Clinician and Client Reports of the Negative Effects of Neuropsychological Assessment for Dementia by Nikki Miller, David J. Grinter, David McGraw, Rachel Pritchett, and Hamish J. McLeod in Journal of Geriatric Psychiatry and Neurology

Supplemental Material - Clinician and Client Reports of the Negative Effects of Neuropsychological Assessment for Dementia Supplemental Material for Clinician and Client Reports of the Negative Effects of Neuropsychological Assessment for Dementia by Nikki Miller, David J. Grinter, David McGraw, Rachel Pritchett, and Hamish J. McLeod in Journal of Geriatric Psychiatry and Neurology
