# Supplementary material for: Clinician and Client Reports of the Negative Effects of Neuropsychological Assessment for Dementia
Source: J Geriatr Psychiatry Neurol. 2025 Dec 26;39(5):638–54. doi: 10.1177/08919887251407122 (PMC13320141; doi:10.1177/08919887251407122)
Supplement: Supplemental Material - Clinician and Client Reports of the Negative Effects of Neuropsychological Assessment for Dementia [file sj-pdf-1-jgp-10.1177_08919887251407122.pdf]

Clinician experiences of neuropsychological assessment questionnaire (v1.1)

This questionnaire contains a number of statements about your experience of providing neuropsychological assessment. The questionnaire will ask you to think about how the assessment impacted your clients and specifically will focus on possible negative effects they experienced. We recognise that clients will likely have had positive experiences of assessment but for the purpose of this questionnaire we would ask you to consider only negative experiences.

Read through the following statements. You will be asked to indicate if, to your knowledge, any of your clients experienced the effect at any point during your assessment. If you answer yes you will be asked to rate the severity of this. You will also be asked at what point during the assessment process your clients experienced this. For this question, please mark all options that apply. You will also be asked if you think the neuropsychological assessment was the most likely cause of the effect in question or if it was more likely due to some other circumstance that was happening in clients' lives at the same time as the assessment. When completing this question, consider all clients you think this impacted and select the option you think was more representative for the majority of your clients. Please put a cross (X) in the appropriate boxes. If you answer no initially to any of the experiences, please move on to the subsequent question. You will also be asked to rate the percentage of patients you have worked with that you think this has been a problem for. Please provide an estimate between 0 to 100%. If you had not ever considered prior to completing this questionnaire that the negative effect in question could arise from neuropsychological assessment, please put a cross in the box in the final column.

You can find an example of how to complete the questionnaire on the following page.

Example completion of questionnaire

	Have any of your clients experienced this before, during or after the assessment?			When it happened, to what extent do you think it affected your client(s)?						At what point in the assessment did they experience this? [Mark all that apply]						In general, what do you think was most likely the cause of this effect?				
	No	Yes	If yes complete next section	Not at all	Slightly	Moderately	Very	Extremely		Before 1st session	During initial interview	During testing	Awaiting feedback	After feedback		The assessment	Other circumstances		Roughly what percentage of your clients do you think this has been a problem for? (0-100%)	Never previously considered effect
1. Stressed	x																			
2. Worried		x				x				x		x	x			x			40%	

Negative effects of neuropsychological assessment for dementia. Participant ID: ________________ Date: ________________

Please answer the following questions:	Have any of your clients experienced this before, during or after the assessment?			When it happened, to what extent do you think it affected your client(s)?					At what point in the assessment did they experience this? [Mark all that apply]					In general, what do you think was most likely the cause of this effect?			
	No	Yes	If yes complete next section	Not at all	Slightly	Moderately	Very	Extremely	Before 1st session	During initial interview	During testing	Awaiting feedback	After feedback	The assessment	Other circumstances	Roughly what percentage of your clients do you think this has been a problem for? (0-100%)	Never previously considered effect
1. Stressed																	
2. Worried																	
3. Hopeless																	
4. Sad																	
5. Disappointment with their performance on the tasks																	
6. Frustrated with themselves																	

Negative effects of neuropsychological assessment for dementia. Participant ID: ________________ Date: ________________

University of Glasgow NHS Lanarkshire

**Please answer the following questions:**	**Have any of your clients experienced this before, during or after the assessment?**			**When it happened, to what extent do you think it affected your client(s)?**						**At what point in the assessment did they experience this? [Mark all that apply]**						**In general, what do you think was most likely the cause of this effect?**				
	**No**	**Yes**	**If yes comple te next section**	**Not at all**	**Slightly**	**Moderately**	**Very**	**Extremely**		**Before 1st session**	**During initial interview**	**During testing**	**Awaiting feedback**	**After feedback**		**The assessment**	**Other circumstances**		**Roughly what percentage of your clients do you think this has been a problem for? (0-100%)**	**Never previously considered effect**
7. Self-critical																				
8. Had thoughts like it would be better if they did not exist anymore" or that they should take their own life																				
9. Irritable																				
10. Angry																				
11. Embarrassed																				
12. Disempowered																				
13. Stupid																				

Negative effects of neuropsychological assessment for dementia. Participant ID: _______________ Date: _______________

University of Glasgow NHS Lanarkshire

**Please answer the following questions:**	**Have any of your clients experienced this before, during or after the assessment?**			**When it happened, to what extent do you think it affected your client(s)?**						**At what point in the assessment did they experience this? [Mark all that apply]**						**In general, what do you think was most likely the cause of this effect?**				
	**No**	**Yes**	**If yes complete next section**	**Not at all**	**Slightly**	**Moderately**	**Very**	**Extremely**		**Before 1st session**	**During initial interview**	**During testing**	**Awaiting feedback**	**After feedback**		**The assessment**	**Other circumstances**		**Roughly what percentage of your clients do you think this has been a problem for? (0-100%)**	**Never previously considered effect**
14. Confused																				
15. Worried about the outcome of the assessment																				
16. Physically tired																				
17. Mentally drained																				
18. Headaches																				
19. Problems with their sleep																				
20. Strain on their family relationships																				

Negative effects of neuropsychological assessment for dementia. Participant ID: ________________ Date: ________________

Please answer the following questions:	Have any of your clients experienced this before, during or after the assessment?			When it happened, to what extent do you think it affected your client(s)?						At what point in the assessment did they experience this? [Mark all that apply]						In general, what do you think was most likely the cause of this effect?				
	No	Yes	If yes comple te next section	Not at all	Slightly	Moderately	Very	Extremely		Before 1st session	During initial interview	During testing	Awaiting feedback	After feedback		The assessment	Other circumstances		Roughly what percentage of your clients do you think this has been a problem for? (0-100%)	Never previously considered effect
21. Lost out financially (e.g. travel costs, loss of employment/wages) to attend appointments																				
22. Had to give up significant amounts of their (and/or carer's) time to attend their appointments																				
23. Lost their driver's license																				

Negative effects of neuropsychological assessment for dementia. Participant ID: _______________ Date: _______________

**Please answer the following questions:**	**Have any of your clients experienced this before, during or after the assessment?**			**When it happened, to what extent do you think it affected your client(s)?**					**At what point in the assessment did they experience this? [Mark all that apply]**					**In general, what do you think was most likely the cause of this effect?**			
	**No**	**Yes**	**If yes complete next section**	**Not at all**	**Slightly**	**Moderately**	**Very**	**Extremely**	**Before 1st session**	**During initial interview**	**During testing**	**Awaiting feedback**	**After feedback**	**The assessment**	**Other circumstances**	**Roughly what percentage of your clients do you think this has been a problem for? (0-100%)**	**Never previously considered effect**
24. Did not understand the purpose of the assessment																	
25. Expressed that they did not understand the results of their assessment																	
26. Expressed that they were not made aware of risks involved in the assessment																	

Negative effects of neuropsychological assessment for dementia. Participant ID: _______________ Date: _______________

University of Glasgow NHS Lanarkshire

Please answer the following questions:	Have any of your clients experienced this before, during or after the assessment?			When it happened, to what extent do you think it affected your client(s)?						At what point in the assessment did they experience this? [Mark all that apply]						In general, what do you think was most likely the cause of this effect?				
	No	Yes	If yes complete next section	Not at all	Slightly	Moderately	Very	Extremely		Before 1st session	During initial interview	During testing	Awaiting feedback	After feedback		The assessment	Other circumstances		Roughly what percentage of your clients do you think this has been a problem for? (0-100%)	Never previously considered effect
27. Expressed that they did not feel prepared for what the assessment would involve																				
28. Expressed that they waited too long to receive feedback from their assessment																				

Additional question:

Describe briefly in your own words any additional negative effects related to the assessment process you think your clients have experienced that have not been covered above:

____

____

____

Please also complete the following questions:

1. **How long have you been qualified as a Clinical Psychologist? ______________**
2. **How long have you been administering neuropsychological assessments? __________________**
3. **How many neuropsychological assessments roughly have you completed? _________________**
4. **What service do you currently work in? ____**
5. **What services have you previously worked in administering neuropsychological assessment? ____**
