# Supplementary material for: Clinician and Client Reports of the Negative Effects of Neuropsychological Assessment for Dementia
Source: J Geriatr Psychiatry Neurol. 2025 Dec 26;39(5):638–54. doi: 10.1177/08919887251407122 (PMC13320141; doi:10.1177/08919887251407122)
Supplement: Supplemental Material - Clinician and Client Reports of the Negative Effects of Neuropsychological Assessment for Dementia [file sj-pdf-2-jgp-10.1177_08919887251407122.pdf]

Client experiences of Neuropsychological assessment questionnaire (v1.1)

This questionnaire contains a number of statements about your recent experience of neuropsychological assessment. The questionnaire will ask you to think about how the assessment impacted you and will focus on any possible negative effects you might have experienced. We recognise that you might have had a positive experience but for the purpose of this questionnaire we would ask you to consider only negative experiences.

Read through the following statements. You will be asked to indicate if you experienced the effect at any point during your assessment. If you answer yes you will be asked to rate the severity of this. You will then be asked when in the assessment process you experienced this. Please mark all options that apply to you. You will also be asked if you think the neuropsychological assessment was the direct cause of this or if it was because of some other circumstance that was happening in your life at the same time as your assessment. Please put a cross (X) in the appropriate boxes. If you answer no to experiencing any of the effects, please move on to the subsequent question.

Example of how to complete the questionnaire

	**Did you experience this before, during or after the assessment?**			**To what extent did this affect you?**					**At what point in the assessment did you experience this? [Mark all that apply]**					**Cause of this negative effect?**	
	**No**	**Yes**	**If yes complete next section**	**Not at all**	**Slightly**	**Moderately**	**Very**	**Extremely**	**Before 1st session**	**During initial interview**	**During testing**	**Awaiting feedback**	**After feedback**	**My assessment**	**Other circumstances**
1. I felt stressed	X														
2. I felt worried		X			X				X		X			X	

**Please answer the following questions:**	**Did you experience this before, during or after the assessment?**			**To what extent did this affect you?**						**At what point in the assessment did you experience this? [Mark all that apply]**						**Cause of this negative effect**	
	**No**	**Yes**	**If yes complete next section**	**Not at all**	**Slightly**	**Moderately**	**Very**	**Extremely**		**Before 1st session**	**During initial interview**	**During testing**	**Awaiting feedback**	**After feedback**		**My assessment**	**Other circumstances**
1. I felt stressed																	
2. I felt worried																	
3. I felt hopeless																	
4. I felt sad																	
5. I was disappointed with my performance on the tasks																	
6. I was frustrated with myself																	
7. I was critical of myself																	
8. I got thoughts like “it would be better if I did not exist anymore” or that “I should take my own life”																	
9. I felt irritable																	
10. I felt angry																	

Negative effects of neuropsychological assessment for dementia Participant ID: ____________________ Date: _________________

**Please answer the following questions:**	**Did you experience this before, during or after the assessment?**			**To what extent did this affect you?**						**At what point in the assessment did you experience this? [Mark all that apply]**						**Cause of this negative effect**	
	**No**	**Yes**	**If yes complete next section**	**Not at all**	**Slightly**	**Moderately**	**Very**	**Extremely**		**Before 1st session**	**During initial interview**	**During testing**	**Awaiting feedback**	**After feedback**		**My assessment**	**Other circumstances**
11. I felt embarrassed																	
12. I felt disempowered																	
13. I felt stupid																	
14. I felt confused																	
15. I worried about the outcome of the assessment																	
16. I felt physically tired																	
17. I felt mentally drained																	
18. I had headaches																	
19. I had problems with my sleep																	
20. I experienced strain on my family relationships																	

Please answer the following questions:	Did you experience this before, during or after the assessment?			To what extent did this affect you?						At what point in the assessment did you experience this? [Mark all that apply]						Cause of this negative effect	
	No	Yes	If yes complete next section	Not at all	Slightly	Moderately	Very	Extremely		Before 1st session	During initial interview	During testing	Awaiting feedback	After feedback		My assessment	Other circumstances
21. I lost out financially (e.g. travel costs, loss of employment/wages) to attend the appointments																	
22. I (or my family member/carer) had to give up significant amounts of time to attend the appointments																	
23. I lost my drivers license																	
24. I did not understand the purpose of the assessment																	
25. I did not understand the results of the assessment																	
26. I was not made aware of risks involved in the assessment																	
27. I did not feel prepared for what the assessment involved																	

**Please answer the following questions:**	**Did you experience this before, during or after the assessment?**			**To what extent did this affect you?**						**At what point in the assessment did you experience this? [Mark all that apply]**						**Cause of this negative effect**	
	**No**	**Yes**	**If yes complete next section**	**Not at all**	**Slightly**	**Moderately**	**Very**	**Extremely**		**Before 1st session**	**During initial interview**	**During testing**	**Awaiting feedback**	**After feedback**		**My assessment**	**Other circumstances**
28. I waited too long to receive feedback from my assessment																	

Additional question:

Describe briefly in your own words whether you experienced any other negative effects directly related to the assessment process that have not been covered above

____

____

____

____

____

____

____

Please also complete the following questions:

1. **What age are you? _______________**
2. **What is your gender? _____________**
3. **What is your ethnicity? ___________**
4. **Did your neuropsychological assessment lead to any diagnoses (e.g mild cognitive impairment, dementia), if yes please specify ________________**
5. **What, if any, physical health conditions have you been diagnosed with? ____**
6. **What, if any, mental health conditions have you been diagnosed with? ____**
