# Supplementary material for: Clinician and Client Reports of the Negative Effects of Neuropsychological Assessment for Dementia
Source: J Geriatr Psychiatry Neurol. 2025 Dec 26;39(5):638–54. doi: 10.1177/08919887251407122 (PMC13320141; doi:10.1177/08919887251407122)
Supplement: Supplemental Material - Clinician and Client Reports of the Negative Effects of Neuropsychological Assessment for Dementia [file sj-pdf-3-jgp-10.1177_08919887251407122.pdf]

University of Glasgow

NHS Lanarkshire

Topic Guide

Participant ID: ____________________ Date of participation: ______________

Demographic information:

1. How long have you been qualified as a Clinical Psychologist? __________________
2. How long have you been administering neuropsychological assessments?

   ____
3. How many neuropsychological assessments roughly have you completed?

   ____
4. What services have you previously worked in administering neuropsychological assessment? ____

Interview

I am interested in clients' experience of neuropsychological assessment, particularly the harms and negative effects they might experience. So, I am interested in hearing your experience of conducting neuropsychological assessment and the impact this has on your clients.

Q1. How long have you been conducting neuropsychological assessments and in what contexts?

- What, in your opinion, is the purpose (or are the purposes) of a neuropsychological assessment?

Q2. What do you think your clients typically want to get out of these kinds of assessments?

- what do you think families and carers would like to get out of the assessments?

Q.3. What do you think the benefits of neuropsychological assessments usually are?

Q.4. Do you do pre-diagnostic counselling? What topics do you tend to cover in this?

- Do you have a discussion with your clients about the pros and cons of assessment and if so, what do you tell them?
- If so, when and how is this incorporated into the assessment process? If not, why not?

- How long has this been part of your practice and how did it come to form part of your practice?

Q5. What, if any, negative experiences have you noticed your client's having, or they report having, during the neuropsychological assessment process?

- what factors contributed to these experiences, do you think?
- how frequently have any of these negative effects occurred?
- prompt for emotional, physical or practical impacts of assessment before, during and after the testing.

Q6. What, if any, negative effects have you noticed your clients experience when they have been provided with feedback from their assessment?

- what do you think contributed to, or caused, this?

Q7. To what extent do you think the client/clinician relationship can have on clients' experience of assessment?

Q8. To what extent do you think clients can experience negative effects due to clinician factors?

Q9. How can clinicians reduce the risks or harms of neuropsychology for patients?

- Do you think there are any other measures, or procedures, that could be put in place to mitigate potential harms to clients?
