# Supplementary material for: Clinician and Client Reports of the Negative Effects of Neuropsychological Assessment for Dementia
Source: J Geriatr Psychiatry Neurol. 2025 Dec 26;39(5):638–54. doi: 10.1177/08919887251407122 (PMC13320141; doi:10.1177/08919887251407122)
Supplement: Supplemental Material - Clinician and Client Reports of the Negative Effects of Neuropsychological Assessment for Dementia [file sj-pdf-4-jgp-10.1177_08919887251407122.pdf]

Table summarising the negative effect percentage responses by question item for clinician and clients

Negative Effects Reported in NPA						
	No N (%)		Yes N (%)		Missing data	
	Clinicians	Clients	Clinicians	Clients	Clinicians	Clients
Q1 Stressed	1 (4%)	3 (25%)	24 (96%)	7 (58.3%)	0 (0%)	2 (16.7%)
Q2 Worried	1 (4%)	2 (16.7%)	24 (96%)	8 (66.7%)	0 (0%)	2 (16.7%)
Q3 Hopeless	11 (44%)	8 (66.7%)	14 (56%)	1 (8.3%)	0 (0%)	3 (25%))
Q4 Sad	8 (32%)	6 (50%)	17 (68%)	3 (25%)	0 (0%)	3 (25%)
Q5 Disappointed in performance on the tasks	0 (0%)	1 (8.3%)	25 (100%)	8 (66.7%)	0 (0%)	3 (25%)
Q6 Frustrated	0 (0%)	1 (8.3%)	25 (100%)	7 (58.3%)	0 (0%)	4 (33.3%)
Q7 Critical of self	0 (0%)	3 (25%)	25 (100%)	5 (41.7%)	0 (0%)	4 (33.3%)
Q8 Suicidal ideation	11 (44%)	8 (66.7%)	13 (52%)	0 (0%)	0 (0%)	4 (33.3%)
Q9 Irritable	6 (24%)	5 (50%)	19 (76%)	2 (16.7%)	0 (0%)	4 (33%)
Q10 Angry	10 (40%)	7 (58.3%)	15 (60%)	1 (8.3%)	0 (0%)	4 (33.3%)
Q11 Embarrassed	4 (16%)	5 (41.7%)	21 (84%)	4 (33.3%)	0 (0%)	3 (25%)
Q12 Disempowered	13 (52%)	6 (50%)	12 (48%)	2 (16.7%)	0 (0%)	4 (33.3%)
Q13 Stupid	4 (16%)	4 (33.3%)	21 (84%)	4 (33.3%)	0 (0%)	4 (33.3%)
Q14 Confused	2 (8%)	5 (41.7%)	23 (92%)	4 (33.3%)	0 (0%)	3 (25%)
Q15 Worried about the outcome	0 (0%)	2 (16.7%)	25 (100%)	10 (83.3%)	0 (0%)	0 (0%)
Q16 Physically tired	2 (8%))	7 (58.3%)	23 (92%)	1 (8.3%)	0 (0%)	4 (33.3%)
Q17 Mentally drained	2 (8%)	5 (41.7%)	23 (92%)	3 (25%)	0 (0%)	4 (33.3%)
Q18 Headaches	13 (52%)	7 (58.3%)	12 (48%)	3 (25%)	0 (0%)	2 (16.7%)
Q19 Problems with sleep	6 (24%)	5 (41.7%)	19 (76%)	3 (25%)	0 (0%)	4 (33.3%)

Negative Effects Reported in NPA						
	No N (%)		Yes N (%)		Missing data	
	Clinicians	Clients	Clinicians	Clients	Clinicians	Clients
Q20 Strain on family relationships	5 (20%)	7 (58.3%)	20 (80%)	1 (8.3%)	0 (0%)	4 (33.3%)
Q21 Lost out financially to attend appointments	16 (64%)	8 (66.7%)	9 (36%)	1 (8.3%)	0 (0%)	3 (25%))
Q22 Gave up significant amounts of time to attend	8 (32%)	7 (58.3%)	17 (68%)	2 (16.7%)	0 (0%)	3 (25%)
Q23 Lost driver's license	6 (24%)	8 (66.7%)	19 (76%)	0 (0%)	0 (0%)	4 (33.3%)
Q24 Did not understand the purpose of assessment	5 (20%)	7 (58.3%)	20 (80%)	1 (8.3%)	0 (0%)	4 (33.3%)
Q25 Did not understand the results of assessment	13 (52%)	7 (58.3%)	12 (48%)	1 (8.3%)	0 (0%)	4 (33.3%)
Q26 Was not made aware of the risks involved in assessment	24 (96%)	5 (41.7%)	1 (4%)	2 (16.7%)	0 (0%)	5 (41.7%)
Q27 Did not feel prepared for what the assessment involved	22 (88%)	7 (58.3%)	3 (12%)	2 (16.7%)	0 (0%)	3 (25%)
Q28 Waited too long to receive feedback	18 (72%)	8 (66.7%)	7 (28%)	1 (8.3%)	0 (0%)	3 (25%)

Table summarising the reported extent the negative effect impacted the client and most likely cause of the effect

Question<br>Total N responses	To what extent did it affect?										Most likely cause of the effect?			
	Not at all		Slightly		Moderately		Very		Extremely		Ax		Other	
	Clin<br>N<br>%	Client<br>N<br>%	Clin<br>N<br>%	Client<br>N<br>%	Clin<br>N<br>%	Client<br>N<br>%	Clin<br>N<br>%	Client<br>N<br>%	Clin<br>N<br>%	Client<br>N<br>%	Clin<br>N<br>%	Client<br>N<br>%	Clin<br>N<br>%	Client<br>N<br>%
**Q1 Stressed**<br>Clinician = 24<br>Client = 7	0<br>0	1*<br>14.28	5<br>20.83	2*<br>28.57	15<br>62.5	4*<br>57.14	2<br>8.33	1*<br>14.28	2<br>8.33	0*<br>0	15<br>62.5	6*<br>>100	8 33.33	0<br>0
**Q2 Worried**<br>Clinician = 24<br>Client =8	0<br>0	0<br>0	5<br>20.83	2<br>25	12<br>50	4<br>50	6<br>25	1<br>12.5	0<br>0	0<br>0	12<br>50	6<br>75	9<br>37.5	0<br>0
**Q3 Hopeless**<br>Clinician = 14<br>Client =1	0<br>0	0<br>0	6<br>42.86	0<br>0	4<br>28.57	0<br>0	3<br>21.43	1<br>100	1<br>7.14	0<br>0	3<br>21.43	1*<br>100	11<br>78.57	1*<br>100
**Q4 Sad**<br>Clinician = 17<br>Client =3	0<br>0	0<br>0	8<br>47.06	0<br>0	8<br>47.06	0<br>0	0<br>0	1<br>33.33	0<br>0	0<br>0	4<br>23.53	1<br>33.33	11<br>64.71	1<br>33.33
**Q5 Disappointed in performance on the tasks**<br>Clinician =25<br>Client =8	0<br>0	0<br>0	8<br>32	2<br>25	14<br>56	3<br>37.5	2<br>8	2<br>25	1<br>4	0<br>0	21<br>84	6<br>75	2<br>8	0<br>0
**Q6 Frustrated**<br>Clinician =25<br>Client =7	0<br>0	0<br>0	7<br>28	3<br>42.86	13<br>52	3<br>42.86	4<br>16	0<br>0	0<br>0	1<br>14.29	15<br>60	6<br>85.71	8<br>32	0<br>0
**Q7 Critical of self**<br>Clinician =25<br>Client =5	0<br>0	0<br>0	7<br>28	1<br>20	12<br>48	3<br>60	5<br>20	1<br>20	0<br>0	0<br>0	11<br>44	4<br>80	11<br>44	0<br>0

Question<br>Total N responses	To what extent did it affect?										Most likely cause of the effect?			
	Not at all		Slightly		Moderately		Very		Extremely		Ax		Other	
	Clin<br>N<br>%	Client<br>N<br>%	Clin<br>N<br>%	Client<br>N<br>%	Clin<br>N<br>%	Client<br>N<br>%	Clin<br>N<br>%	Client<br>N<br>%	Clin<br>N<br>%	Client<br>N<br>%	Clin<br>N<br>%	Client<br>N<br>%	Clin<br>N<br>%	Client<br>N<br>%
**Q8 Suicidal ideation**<br>Clinician = 13<br>Client =0	3<br>23.08	N/A	4<br>30.77	N/A	0<br>0	N/A	5<br>38.46	N/A	2<br>15.38	N/A	12<br>92.31	1*<br>>100	0	N/A
**Q9 Irritable**<br>Clinician =19<br>Client =2	0<br>0	0<br>0	8<br>42.11	1<br>50	9<br>47.37	1<br>50	1<br>5.26	0<br>0	0<br>0	0<br>0	8<br>42.11	2*<br>100	10<br>52.63	1*<br>50
**Q10 Angry**<br>Clinician =15<br>Client =1	0<br>0	0<br>0	8<br>53.33	0<br>0	3<br>20	1<br>100	3<br>20	0<br>0	10<br>66.67	0<br>0	8<br>53.33	1*<br>100	6<br>40	1*<br>100
**Q11 Embarrassed**<br>Clinician =21<br>Client =4	0<br>0	0<br>0	7<br>33.33	5*<br>>100	11<br>52.38	2*<br>50	2<br>9.52	1*<br>25	0<br>0	0<br>0	16<br>76.19	3*<br>75	3<br>14.29	2*<br>50
**Q12 Disempowered**<br>Clinician =12<br>Client =2	0<br>0	0<br>0	7<br>58.33	1<br>50	4<br>33.33	0<br>0	1<br>8.33	1<br>50	0<br>0	0<br>0	7<br>58.33	1<br>50	6<br>50	1<br>50
**Q13 Stupid**<br>Clinician =21<br>Client =4	0<br>0	0<br>0	8<br>38.10	2<br>50	11<br>52.38	1<br>25	1<br>4.76	0<br>0	0<br>0	1<br>25	18<br>85.71	3<br>75	2<br>9.52	0<br>0
**Q14 Confused**<br>Clinician =23<br>Client =4	0<br>0	0<br>0	8<br>34.78	2<br>50	8<br>34.78	0<br>0	4<br>17.39	1<br>25	1<br>4.35	0<br>0	9<br>39.13	3<br>75	12<br>52.17	0<br>0

Question<br>Total N responses	To what extent did it affect?										Most likely cause of the effect?			
	Not at all		Slightly		Moderately		Very		Extremely		Ax		Other	
	Clin<br>N<br>%	Client<br>N<br>%	Clin<br>N<br>%	Client<br>N<br>%	Clin<br>N<br>%	Client<br>N<br>%	Clin<br>N<br>%	Client<br>N<br>%	Clin<br>N<br>%	Client<br>N<br>%	Clin<br>N<br>%	Client<br>N<br>%	Clin<br>N<br>%	Client<br>N<br>%
**Q15 Worried about the outcome**<br>Clinician =25<br>Client =10	0<br>0	0<br>0	4<br>16	1<br>10	9<br>36	3<br>30	10<br>40	1<br>10	0<br>0	1<br>10	15<br>60	3<br>30	6<br>24	0<br>0
**Q16 Physically tired**<br>Clinician =23<br>Client =1	0<br>0	1<br>100	8<br>34.78	0<br>0	10<br>43.48	0<br>0	2<br>8.70	0<br>0	1<br>4.35	0<br>0	10<br>43.48	0<br>0	10<br>43.48	0<br>0
**Q17 Mentally drained**<br>Clinician =23<br>Client =3	0<br>0	0<br>0	9<br>39.13	1<br>33.33	7<br>30.43	1<br>33.33	5<br>21.74	0<br>0	0<br>0	1<br>33.33	14<br>60.87	2<br>66.67	0<br>0	0<br>0
**Q18 Headaches**<br>Clinician =12<br>Client =3	0<br>0	1<br>33.33	8<br>66.67	1<br>33.33	3<br>25	0<br>0	1<br>8.33	0<br>0	0<br>0	0<br>0	3<br>25	1<br>33.33	9<br>75	0<br>0
**Q19 Problems with sleep**<br>Clinician =19<br>Client =3	2<br>10.53	0<br>0	4<br>21.05	2<br>66.67	9<br>46.37	0<br>0	3<br>15.79	1<br>33.33	0<br>0	0<br>0	1<br>5.26	2<br>66.67	15<br>78.95	0<br>0
**Q20 Strain on family relationships**<br>Clinician =20<br>Client =1	0<br>0	0<br>0	4<br>20	0<br>0	8<br>40	0<br>0	7<br>35	0<br>0	0<br>0	1<br>100	3<br>15	0<br>0	17<br>85	1<br>100

	To what extent did it affect?										Most likely cause of the effect?			
	Not at all		Slightly		Moderately		Very		Extremely		Ax		Other	
**Question**<br>Total N responses	**Clin<br>N<br>%**	**Client<br>N<br>%**	**Clin<br>N<br>%**	**Client<br>N<br>%**	**Clin<br>N<br>%**	**Client<br>N<br>%**	**Clin<br>N<br>%**	**Client<br>N<br>%**	**Clin<br>N<br>%**	**Client<br>N<br>%**	**Clin<br>N<br>%**	**Client<br>N<br>%**	**Clin<br>N<br>%**	**Client<br>N<br>%**
**Q21 Lost out financially to attend appointments**<br>Clinician =9<br>Client =1	0<br>0	1*<br>100	6<br>66.67	0<br>0	1<br>11.11	2*<br>>100	0<br>0	0<br>0	0<br>0	0<br>0	7<br>77.78	1<br>100	2<br>22.22	0<br>0
**Q22 Gave up significant amounts of time to attend**<br>Clinician =17<br>Client =2	0<br>0	0<br>0	8<br>47.06	0<br>0	6<br>35.29	1<br>50	1<br>5.88	1<br>50	0<br>0	0<br>0	16<br>94.12	0<br>0	1<br>5.88	1<br>50
**Q23 Lost drivers license**<br>Clinician =19<br>Client =0	0<br>0	N/A	0<br>0	N/A	2<br>10.53	N/A	6<br>31.58	N/A	4<br>21.05	N/A	10<br>52.63	N/A	5<br>26.36	N/A
**Q24 Did not understand the purpose of assessment**<br>Clinician =20<br>Client =1	1<br>5	0<br>0	6<br>30	0<br>0	5<br>25	0<br>0	4<br>20	1<br>100	0<br>0	0<br>0	2<br>10	0<br>0	15<br>75	1<br>100

	To what extent did it affect?										Most likely cause of the effect?			
	Not at all		Slightly		Moderately		Very		Extremely		Ax		Other	
**Question**<br>Total N responses	**Clin**<br>**N**<br>**%**	**Client**<br>**N**<br>**%**	**Clin**<br>**N**<br>**%**	**Client**<br>**N**<br>**%**	**Clin**<br>**N**<br>**%**	**Client**<br>**N**<br>**%**	**Clin**<br>**N**<br>**%**	**Client**<br>**N**<br>**%**	**Clin**<br>**N**<br>**%**	**Client**<br>**N**<br>**%**	**Clin**<br>**N**<br>**%**	**Client**<br>**N**<br>**%**	**Clin**<br>**N**<br>**%**	**Client**<br>**N**<br>**%**
**Q25 Did not understand the results of assessment**<br>Clinician =12<br>Client =1	0<br>0	1<br>100	3<br>25	0<br>0	5<br>41.67	0<br>0	0<br>0	0<br>0	0<br>0	0<br>0	1<br>8.33	0<br>0	8<br>66.67	0<br>0
**Q26 Was not made aware of the risks involved in assessment**<br>Clinician =1<br>Client =2	0<br>0	0<br>0	0<br>0	1<br>50	1<br>100	1<br>50	0<br>0	1<br>50	0<br>0	0<br>0	0<br>0	1<br>50	1<br>100	0<br>0
**Q27 Did not feel prepared for what the assessment involved**<br>Clinician =3<br>Client =2	0<br>0	0<br>0	1<br>33.33	1<br>50	2<br>66.67	1<br>50	0<br>0	0<br>0	0<br>0	1<br>50	2<br>66.67	0<br>0	1<br>33.33	2<br>100
**Q28 Waited too long to receive feedback**<br>Clinician =7<br>Client =1	0<br>0	0<br>0	2<br>28.57	0<br>0	3<br>42.86	1*<br>100	1<br>14.29	1*<br>100	0<br>0	0<br>0	5<br>71.43	1*<br>100	1<br>14.29	1*<br>100

Note: Data that is suspected to have been impacted by reporting errors is highlighted by *
Abbreviations: Clin, Clinician

Table summarising at what point during the NPA negative effects were experienced

	At what point in the assessment was this experienced?									
	Before 1st appointment		During initial interview		During testing		Awaiting feedback		After feedback	
**Question**<br>Total N responses	**Clinician N %**	**Client N %**	**Clinician N (%)**	**Client N (%)**	**Clinician N (%)**	**Client N (%)**	**Clinician N (%)**	**Client N (%)**	**Clinician N (%)**	**Client N (%)**
**Q1 Stressed**<br>Clinician =24<br>Client =7	18<br>75	2*<br>28.57	18<br>75	4*<br>57.14	21<br>87.5	1*<br>14.29	17<br>70.83	2*<br>28.57	8<br>33.33	0*<br>0
**Q2 Worried**<br>Clinician =24<br>Client =8	20<br>83.33	4<br>50	20<br>83.33	0<br>0	19<br>79.17	4<br>50	21<br>87.5	3<br>37.5	10<br>41.67	0<br>0
**Q3 Hopeless**<br>Clinician = 14<br>Client =1	9<br>64.29	2*<br>>100	7<br>50	0<br>0	8<br>57.14	0<br>0	5<br>35.71	0<br>0	6<br>42.86	0<br>0
**Q4 Sad**<br>Clinician =17<br>Client =3	8<br>47.06	1<br>33.33	10<br>58.82	2<br>66.67	5<br>29.41	1<br>33.33	3<br>17.65	0<br>0	11<br>64.71	0<br>0
**Q5 Disappointed in performance on the tasks**<br>Clinician =25<br>Client =8	0<br>0	0<br>0	0<br>0	1<br>12.5	25<br>100	7<br>87.5	12<br>48	0<br>0	6<br>24	0<br>0
**Q6 Frustrated**<br>Clinician =25<br>Client =7	6<br>24	0<br>0	7<br>28	0<br>0	25<br>100	6<br>85.71	8<br>32	1<br>14.29	3<br>12	0<br>0
**Q7 Critical of self**<br>Clinician =25<br>Client =5	7<br>28	0<br>0	13<br>52	1<br>20	24<br>96	4<br>80	10<br>40	0<br>0	3<br>12	0<br>0

Question<br>Total N responses	At what point in the assessment was this experienced?<br>Before 1st appointment		During initial interview		During testing		Awaiting feedback		After feedback	
	Clinician N %	Client N %	Clinician N (%)	Client N (%)	Clinician N (%)	Client N (%)	Clinician N (%)	Client N (%)	Clinician N (%)	Client N (%)
**Q8 Suicidal ideation**<br>Clinician =13<br>Client =0	11<br>84.62	N/A	4<br>30.77	N/A	2<br>15.38	N/A	4<br>30.77	N/A	5<br>38.46	N/A
**Q9 Irritable**<br>Clinician =19<br>Client =2	11<br>57.89	1<br>50	14<br>73.68	1<br>50	15<br>78.95	0<br>0	8<br>42.11	0<br>0	8<br>42.11	0<br>0
**Q10 Angry**<br>Clinician =15<br>Client =1	8<br>53.33	1<br>100	12<br>80	0<br>0	10<br>66.67	0<br>0	5<br>33.33	0<br>0	8<br>53.33	0<br>0
**Q11 Embarrassed**<br>Clinician =21<br>Client =4	4<br>19.05	1<br>25	10<br>47.62	3<br>75	19<br>90.48	4<br>100	3<br>14.29	3<br>75	6<br>28.57	0<br>0
**Q12 Disempowered**<br>Clinician =12<br>Client =2	8<br>66.67	0<br>0	7<br>58.33	1<br>50	6<br>50	0<br>0	4<br>33.33	1<br>50	3<br>25	0<br>0
**Q13 Stupid**<br>Clinician =21<br>Client =4	8<br>38.10	0<br>0	9<br>42.86	0<br>0	19<br>90.48	3<br>75	6<br>28.57	1<br>25	1<br>4.76	0<br>0
**Q14 Confused**<br>Clinician =23<br>Client =4	16<br>69.57	0<br>0	15<br>65.22	0<br>0	18<br>78.26	3<br>75	10<br>43.48	0<br>0	9<br>39.13	0<br>0
**Q15 Worried about the outcome**<br>Clinician =25<br>Client =10	22<br>88	2<br>20	20<br>80	2<br>20	24<br>96	3<br>30	24<br>96	3<br>30	7<br>28	0<br>0

Question<br>Total N responses	At what point in the assessment was this experienced?<br>Before 1st appointment		During initial interview		During testing		Awaiting feedback		After feedback	
	Clinician<br>N<br>%	Client<br>N<br>%	Clinician<br>N<br>(%)	Client<br>N<br>(%)	Clinician<br>N<br>(%)	Client<br>N<br>(%)	Clinician<br>N<br>(%)	Client<br>N<br>(%)	Clinician<br>N<br>(%)	Client<br>N<br>(%)
**Q16 Physically tired**<br>Clinician =23<br>Client =1	7<br>30.43	0<br>0	11<br>47.83	0<br>0	20<br>86.96	0<br>0	22<br>95.65	0<br>0	5<br>21.74	0<br>0
**Q17 Mentally drained**<br>Clinician =23<br>Client =3	4<br>17.39	0<br>0	9<br>39.13	0<br>0	21<br>91.30	2<br>66.67	3<br>13.04	0<br>0	5<br>21.74	0<br>0
**Q18 Headaches**<br>Clinician =12<br>Client =3	6<br>50	0<br>0	5<br>41.67	0<br>0	11<br>91.67	0<br>0	6<br>50	1<br>33.33	5<br>41.67	0<br>0
**Q19 Problems with sleep**<br>Clinician =19<br>Client =3	12<br>63.16	0<br>0	12<br>63.16	0<br>0	13<br>68.42	0<br>0	9<br>47.37	1<br>33.33	10<br>52.63	0<br>0
**Q20 Strain on family relationships**<br>Clinician =20<br>Client =1	20<br>100	0<br>0	19<br>95	0<br>0	11<br>55	1<br>100	15<br>75	0<br>0	14<br>70	0<br>0
**Q21 Lost out financially to attend appointments**<br>Clinician =9<br>Client =1	3<br>33.33	1<br>100	6<br>66.67	0<br>0	7<br>77.78	1<br>100	3<br>33.33	1<br>100	2<br>22.22	1<br>100
**Q22 Gave up significant amounts of time to attend**<br>Clinician =17<br>Client =2	6<br>35.29	0<br>0	11<br>64.71	0<br>0	14<br>82.35	1<br>50	5<br>29.41	0<br>0	7<br>41.17	1<br>50
**Q23 Lost driver's license**<br>Clinician =19<br>Client =0	5<br>26.31	N/A	1<br>5.26	N/A	2<br>10.53	N/A	2<br>10.53	N/A	13<br>68.42	N/A

Question<br>Total N responses	At what point in the assessment was this experienced?<br>Before 1st appointment		During initial interview		During testing		Awaiting feedback		After feedback	
	Clinician<br>N<br>%	Client<br>N<br>%	Clinician<br>N<br>(%)	Client<br>N<br>(%)	Clinician<br>N<br>(%)	Client<br>N<br>(%)	Clinician<br>N<br>(%)	Client<br>N<br>(%)	Clinician<br>N<br>(%)	Client<br>N<br>(%)
**Q24 Did not understand the purpose of assessment**<br>Clinician =20<br>Client =1	15<br>75	1<br>100	15<br>75	0<br>0	7<br>35	0<br>0	1<br>5	0<br>0	1<br>5	0<br>0
**Q25 Did not understand the results of assessment**<br>Clinician =12<br>Client =1	0<br>0	1<br>100	0<br>0	0<br>0	0<br>0	0<br>0	2<br>16.67	0<br>0	11<br>91.67	0<br>0
**Q26 Was not made aware of the risks involved in assessment**<br>Clinician =1<br>Client =2	1<br>100	2<br>100	1<br>100	1<br>50	0<br>0	0<br>0	0<br>0	0<br>0	0<br>0	0<br>0
**Q27 Did not feel prepared for what the assessment involved**<br>Clinician =3<br>Client =2	1<br>33.33	1<br>50	2<br>66.67	1<br>50	2<br>66.67	1<br>50	1<br>33.33	1<br>50	0<br>0	0<br>0
**Q28 Waited too long to receive feedback**<br>Clinician =7<br>Client =1	2<br>28.57	0<br>0	1<br>14.29	0<br>0	1<br>14.29	1<br>100	6<br>85.71	0<br>0	3<br>42.86	0<br>0

Note: Data that is suspected to have been impacted by reporting errors is highlighted by *
